# Fracture Failure Mechanisms of Long Single PA6 Fibers

**DOI:** 10.3390/polym9070243

**Published:** 2017-06-23

**Authors:** Huiling Ding, Yongzhen Zhang, Zhitao He

**Affiliations:** 1 School of Mechanical Engineering, Northwestern Polytechnical University, Xi’an 710000, China; ding-huiling@163.com; 2 School of Agricultural Equipment Engineering, Henan University of Science and Technology, Luoyang 471023, China; hezt79@163.com; 3 School of Materials Science and Engineering, Henan University of Science and Technology, Luoyang 471023, China

**Keywords:** single fiber, cutting, fracture morphology, failure mechanism

## Abstract

The present study investigates the failure mechanisms of industrial fiber materials, using a custom designed fiber cutting performance test bench. The fracture morphologies of single PA6 fibers are examined by scanning electron microscopy. The analysis reveals that fiber cutting can be distinguished according to four distinct stages of fiber failure represented by shearing, cutting, brittle fracture, and tensile failure, which are the result of different mechanisms active during the processes of crack initiation, extension, and fracture. The results of fractographic analysis are further verified by an analysis of the blade assembly speed with respect to time over the entire fracture failure process based on high-speed camera data. The results of fractographic analysis and blade assembly speed are fully consistent.

## 1. Introduction

Natural and synthetic fibers can be employed to create materials with high strength, high elastic modulus, light weight, and low cost. Fibers play an irreplaceable role in many industries such as national defense, aviation, aerospace, and construction. Among synthetic fibers, the PA6 polycrystalline polyimide fiber is one of the most widely employed polymer fibers, and has been adopted in an extensive variety of applications owing to its good strength and durability. Synthetic polyamides consist of aliphatic polyamides and aromatic polyamides. The aliphatic family of polyamides includes the well-known thermoplastic nylon. Nylon is one of the most important engineering plastics, and forms the basis for the PA6 fiber. The majority of commercially available synthetic polyamide polymer fibers and PA6 in particular have been applied in filtration and life sciences [1]. Reinforcement with PA6 fiber is one of the most effective methods of improving the properties of cement-based materials [2]. PA6 fiber has been the subject of extensive research, including its manufacturing processes, mechanical properties, and the effect of embedded materials. Kailash and Tippur [3] found that composites with a fiber volume ratio of 3% PA6 filler produced an equivalent dynamic fracture toughness enhancement as composites with a fiber volume ratio of 10% glass. The cutting performance of a PA6 fiber is one of its most important mechanical properties. Unfortunately, even though PA6 fiber has been widely employed, little work has been conducted for investigating the cutting mechanisms of PA6 fibers.

The nature of fibrous materials makes them ideally suited to the manufacture of continuous sheets that must be subsequently cut into the specific shapes required by industry, which consumes considerable energy. As such, considerable effort has been expended to reduce the power consumption of the cutting process by way of lowering cutting resistance based on the cutting mechanics of fibrous materials [4,5,6,7]. For example, Dowgiallo [6] developed a rheological cutting energy model derived from experimental cutting tests conducted for four different fibrous materials. Kakitis et al. [7] conducted experimental cutting tests on six types of hemp fibers using a rotating knife. The researchers developed an interaction model between the fiber and cutter, and proposed that the specific cutting energy depended on the extent of rope twisting. In addition, fibrous materials are also commonly required to have extremely high resistance to cutting and fracture failure. As a result, considerable effort has also been expended to enhance the cutting resistance of fibrous materials [8,9,10,11,12,13]. For example, Kothari et al. [8] developed a mathematical model for predicting the cutting resistance of woven cotton textiles. Vu Thi et al. [9] studied the mechanics and mechanisms of cutting resistance for protective materials in the presence of friction. Mayo and Wetzel [10] developed a model reflecting the static stress failure of fibers based on experimental cutting resistance and failure results collected for six high-performance single fibers. Kane et al. [11] studied the effects of impact force and rope tension on the cutting resistance of a climbing rope cut with a handsaw.

The above summary indicates that considerable research has been conducted to evaluate the general forces involved with cutting resistance, and predictive models have been developed accordingly. However, we note that the cutting resistance of fibrous materials is generally based on the fracture mechanics of the individual fibers. Unfortunately, little work has been conducted from this perspective. The processes of crack initiation, extension, and fracture in individual fibers provide an accurate record of the conditions encountered during the cutting of fibrous materials. Therefore, qualitative analyses of the fracture mechanics of individual fibers will provide important evidence for developing fracture failure models and supply vital clues for diagnosing the causes of fracture failure and the mechanisms affecting cutting resistance.

To facilitate the above stated goals, this paper investigates the fracture mechanics of single PA6 fibers using a custom designed fiber cutting performance test bench. The fracture morphologies of the PA6 fiber are examined by scanning electron microscopy (SEM). Fractographic studies demonstrate the occurrence of different mechanisms active during the process of crack initiation, extension, and fracture. In addition, the fracture failure processes were recorded by high-speed camera, and a blade assembly speed variation curve is obtained. The analyses reveal four distinct fracture failure mechanisms active at different periods of the single fiber cutting process. This paper provides much needed insight into the cutting mechanisms of widely employed PA6 fibers, and is expected to contribute to an understanding of cutting fracture failure mechanisms useful to the fields of polymer fiber science and engineering.

## 2. Materials and Methods

### 2.1. Test Materials

The material properties of PA6 fibers vary according to the manufacturing technique employed in their production, and a number of manufacturing techniques have been developed [14,15,16]. Zhang et al. [14] successfully produced fine-denier PA6 fibers using additives containing lanthanide compounds, and the crystallization and phase transition of the resulting PA6 fibers were investigated during spinning and drawing processes. Polyvinylidene fluoride (PVDF) fiber mats with average fiber diameters of 200–2000 nm were fabricated under controlled electro-spinning conditions [16]. The testing conducted in this work employed PA6 (polyamide Nylon 6) trimmer line monofilament fabricated by slice spinning (Nantong NTEC Monofilament Technology Co., Ltd., Nantong, China). The original trimmer line material was 450 mm in length and 3 mm in diameter. The mechanical properties of the PA6 employed in testing are listed in Table 1.

### 2.2. Test Bench

**Figure 1:**
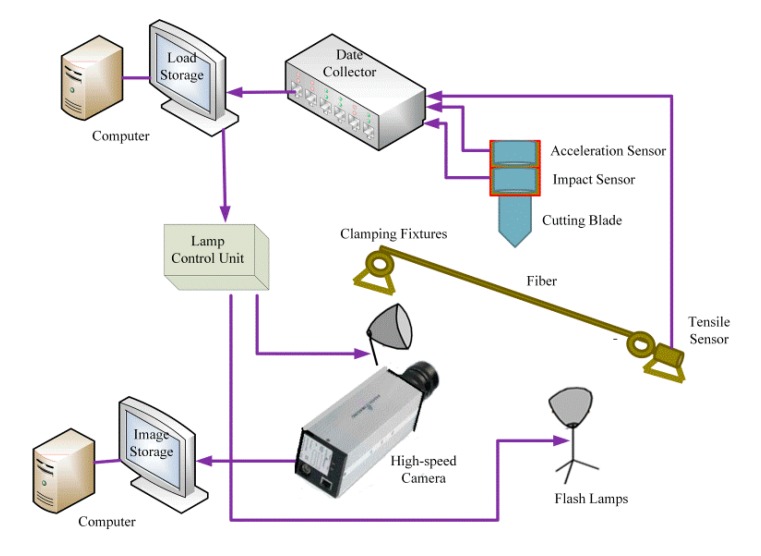
Schematic of the PA6 monofilament gravity cutting test bench.

Figure 1 presents a schematic of the custom-designed test bench employed for evaluation of fiber cutting. The bench mainly includes two clamping fixtures, cutting blade control, and a test system. The gauge length *L*_0_ between the two clamping fixtures was 220 mm. The test system mainly includes an impact sensor and acceleration sensor installed on the top of the cutting blade and a tensile stress sensor installed on one of the clamping fixtures. The sensors can measure the forces acting on the fiber during cutting in real time. A high-speed camera was employed to record images of the cutting process in real-time at a rate of 10,000 frames per second (fps).

**Figure 2:**
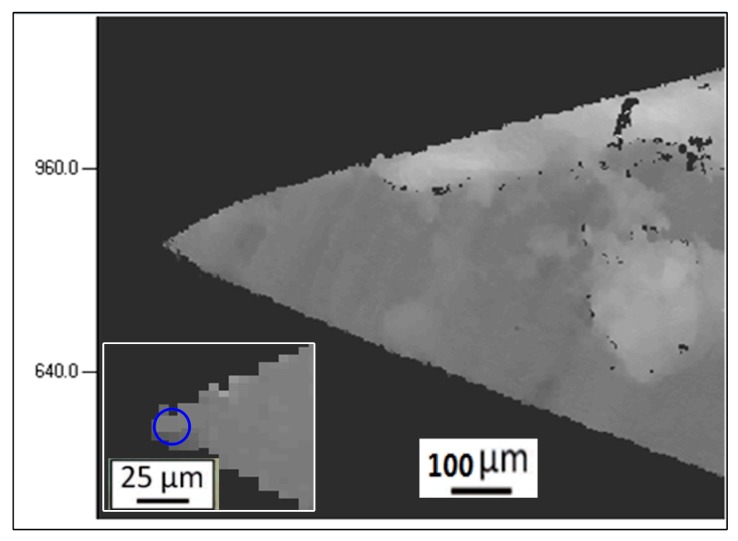
Representative optical micrograph of the blade tip showing an average edge radius of 15.1 ± 0.5 μm.

The mass of the cutting blade assembly can be varied to evaluate its effect on cutting performance, although only a single value of *m* was considered in the present work (i.e., 2 kg). The cutting blade consisted of high manganese steel with a blade tip angle of 30°. Figure 2 shows a representative optical micrograph of the blade tip. The radius of the blade tip had an average value *r* = 15.1 ± 0.5 μm based on measurements conducted using three similar images. Here, we note that the sharpness of the blade increases with decreasing *r*. The cutting blade was positioned orthogonally to the fiber during all tests.

### 2.3. Test Process

**Figure 3:**
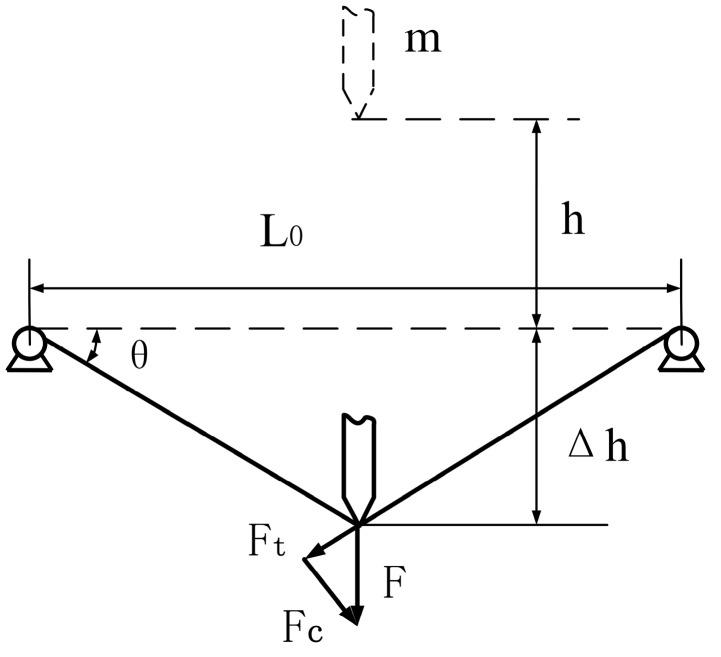
Conditions of fiber-blade contact at fiber failure with a 90° slice angle, showing a initial distance *h*, a vertical deflection distance Δ*h*, a fiber deflection angle θ, and tensile and compressive force components *F*_t_ and *F*_c_, respectively.

To mount a fiber for testing, one end of the fiber is clamped in the test fixture, and then the fiber is pre-tensioned to 10% of its maximum tensile strength using a static weight attached to the other end of the fiber. The maximum tensile force was between 60 and 80 N in this study, and the value of the pre-tightening force was 6–8 N. While under tension, the second fiber end is clamped in the test fixture. With a single fiber mounted in the test fixture, the cutting blade assembly with a rest mass of *m* was raised to a height *h* above the midpoint of the fiber and released at zero initial speed. The cutting blade assembly then fell vertically along the cylindrical guide rails under the force of gravity to cut the filament. The effect of friction was reduced to a negligible concern by installing linear bearings between the cutting blade assembly and guide rails. Therefore, friction in the apparatus was ignored during testing. Figure 3 illustrates the geometry of the cutting process for a 90° slice angle, where the value of *L*_0_ was 220 mm, *h* was preset in accordance with the requirements of the experiment, θ is the angle of fiber deflection, and Δ*h* represents the vertical deflection of the fiber, which resolves the cutting force *F* into tensile force *F*_t_ and compressive force *F*_c_ components.

Because the PA6 fibers employed in this study are anisotropic, slight and uncontrollable variations in the blade edge geometry could have a relatively substantial impact on the fracture results obtained. Therefore, all cutting tests were conducted five times to ensure an adequate reproducibility of the experimental results under equivalent conditions, and the reported blade assembly speed and fiber stress results are the average values obtained for at least three identical tests. The characteristics of fiber failure were examined by SEM using a JSM-5610LV (JEOL, Tokyo, Japan) field emission scanning electron microscope with an accelerating voltage of 20 kV. We note that polymeric materials are very sensitive to electron beam damage [16,17,18]. However, in their study of PVDF molecules in nanofibers imaged at an atomic scale by aberration-corrected electron microscopy, Lolla et al. [17] found that the sensitivity of fibers to electron radiation damage increased with decreasing fiber diameter. The PA6 fibers employed in the present study had relatively large diameters and very low conductivity. Therefore, to reduce the possibility of electron beam damage and increase the surface conductivity of PA6 fibers, all fibers (after failure) were sputtered with a few nanometers of silver using a JFC-1600 auto fine coater (JEOL, Tokyo, Japan). Therefore, we conclude that the SEM results were largely unaffected by electron beam damage.

## 3. Results and Discussion

**Figure 4:**
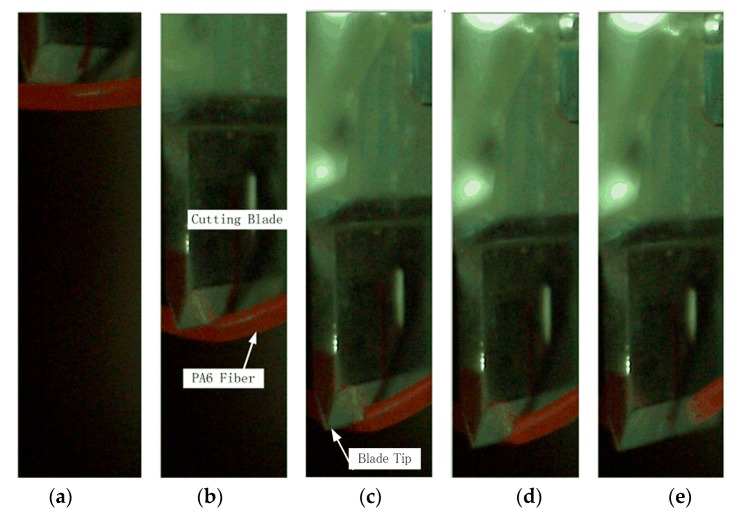
Images of the cutting process obtained by high-speed camera at different times: (**a**) the blade makes initial contact with the PA6 fiber; (**b**) the blade tip compresses the fiber; (**c**) the blade tip begins cutting into the fiber; (**d**) subsequent analysis shows that the fiber undergoes brittle fracture at this point of the cutting process; (**e**) the blade completely severs the fiber.

Based on the nature of the cutting apparatus employed, the cutting process can be divided into three distinct stages: the initial stage, compression stage, and fiber failure stage. Figure 4 presents representative images of the cutting process obtained by high-speed camera at different times.

### 3.1. Initial Stage

Prior to making contact with the fiber, the cutting blade assembly is in an approximate free fall condition. When the blade initially contacts the fiber, the fiber is subject to an impact force, and axial elastic stretching occurs. The fiber and blade assembly initially drop synchronously with negligible interactive force between the two. Here, relative to the value of *m*, the mass of the fiber can be ignored, and the blade assembly speed is not affected to a great extent in this stage.

### 3.2. Compression Stage

With increasing tension, the rate of axial elastic stretching decreases, producing an increasing upward resistance force between the fiber and blade, which correspondingly reduces the blade assembly speed. Conversely, the blade produces an increasing downward compressive radial stress on the fiber. Under this condition, the fiber is subject to both increasing tensile stress and compressive stress, which eventually reaches the fatigue limit of the fiber, whereupon the fiber failure stage begins.

### 3.3. Fiber Failure Stage

**Figure 5:**
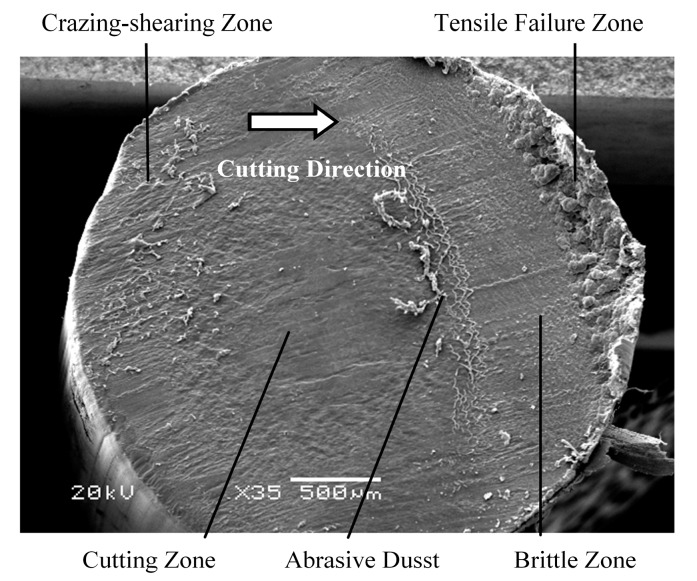
SEM image of a representativefractured fiber end presenting four distinct fracture zones.

As shown in Figure 5, a representative fractured fiber end presents four distinct fracture zones, denoted as the craze-shearing zone, cutting zone, brittle-fracture zone, and tensile-failure zone, with obvious borders between them. These areas reflect distinct fracture mechanisms associated with crack initiation, crack extension, and fiber failure.

#### 3.3.1. Crack Initiation—Craze-Shearing Zone

The action of the cutting blade on the fiber develops a local transverse compressive stress on the fiber at the blade tip. For a fiber under tension, as in the present experimental configuration, this transverse compression is likely to induce local flaws in the fiber, such as cracks, that then lead to progressive local tensile failure. For a cutting blade acting with a force *F* at normal incidence on a fiber of diameter *D*, the compressive stress induced by the blade σ_B_ is given approximately by σ_B_ = *F*_c_/(2*r*β*D*), where β is a factor employed to compensate for secondary effects, such as fiber cross-sectional deformation owing to compression by the section of the fiber, and as the fiber deforms blade under loading. A detailed analysis of the changes in the fiber cross section induced by the progression of the blade through the fiber is exceedingly difficult. Therefore, we assume β = 1 in the present study, as has been done previously [7]. In response to increasing σ_B_ on the fiber and increasing fiber tension with the continued downward motion of the blade assembly, the blade tip forms an indentation on the fiber surface owing to elastic deformation. The depth of the indentation increases with increasing σ_B_. Under this condition, the fiber on both sides of the blade tip is stretched to the elastic limit, and plastic deformation occurs. Further increase in σ_B_ gives rise to a large number of crazing and shearing belts within the fiber. This produces an obvious shear yielding, while the fiber on both sides of the blade tip also undergoes craze yielding due to increased tensile stress. This process induces the formation of cracks at the point of highest σ_B_ under the blade tip.

**Figure 6:**
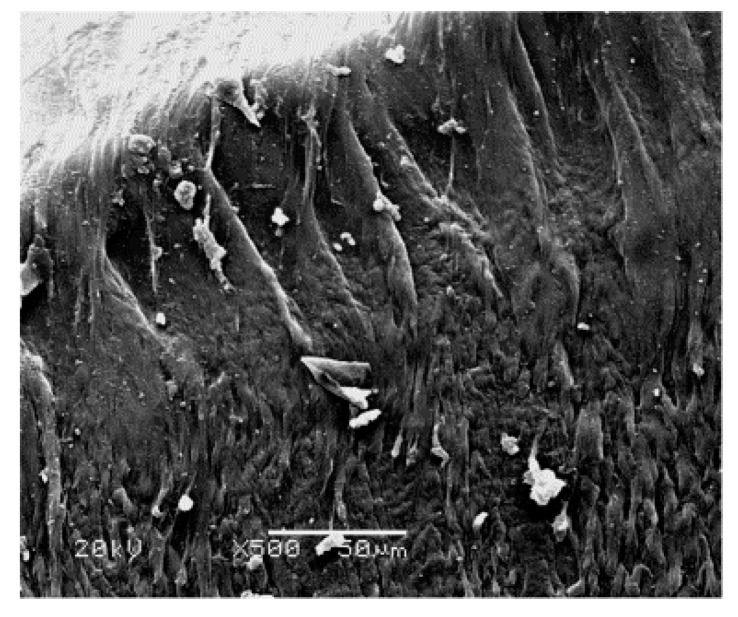
SEM image of the craze-shearing zone shown in Figure 5.

Figure 6 presents a magnified SEM image of the craze-shearing zone shown in Figure 5. Here, smooth fiber strands representative of craze fibrils are observed at the edge of the fiber fracture surface. Plastic deformation of the craze fibrils is severe, and is accompanied with scattered fiber fragments of various shapes and sizes. The fibrils in the craze-shearing zone are severely stretched to a final length of about 100 μm, torn, and broken. The destruction is mainly caused by shear yielding.

#### 3.3.2. Crack Extension—Cutting Zone

The compressive stress sustained by the fiber is perpendicular to the plane where the cracks initially form. As the compressive and tensile stresses of the fiber increase, the blade travels through the fiber along the initial cracks, forming a cutting section. At this point in the fracture process, frictional forces act on the fiber in the same direction as that of crack extension. Thus, the fiber material is stretched and undergoes plastic deformation due to friction.

**Figure 7:**
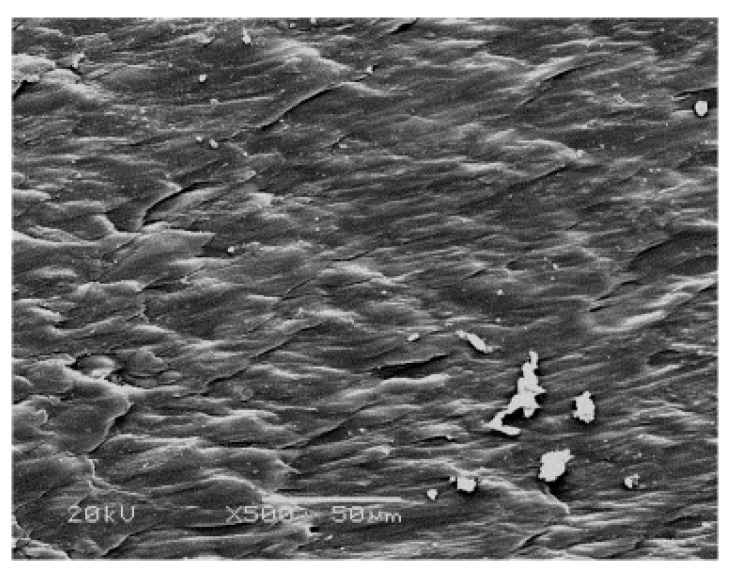
SEM image of the cutting zone shown in Figure 5.

Figure 7 presents a magnified SEM image of the cutting zone shown in Figure 5. We note that the cutting cross section is relatively smooth, and the plastic deformation due to friction produces a uniform distribution of stretched and broken features. We also note that the fibrils are stretched to a final length of about 50 μm, the direction of plastic deformation is the same as that of crack extension, and fiber fragments are sporadically scattered within the cutting zone.

#### 3.3.3. Rapid Crack Extension—Brittle-Fracture Zone

As the blade travels through the fiber, the effective cross-sectional area of the fiber decreases while, simultaneously, the tension in the fiber increases due to the continued downward motion of the cutting assembly. Eventually, the tensile stress exceeds the critical tensile stress of the fiber, and cracks extend rapidly. At this point of the fracture process, the speed of crack extension is greater than the speed of the cutting blade. Therefore, the blade is not in contact with the fiber, and zero frictional force is applied to both sides of the cutting blade. Under the high frictional resistance in the cutting zone, considerable debris accumulates on the cutting blade, which is then deposited on the cutting face when fracture enters the rapid crack expansion stage. This explains the obvious line of demarcation between the cutting zone and brittle-fracture zone observed in Figure 5.

**Figure 8:**
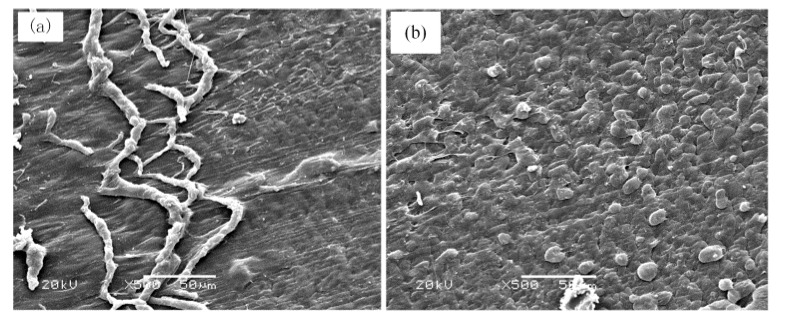
SEM images of the area of abrasive dust accumulation (**a**) and the brittle-fracture zone (**b**) shown in Figure 5.

Figure 8a,b respectively presents magnified SEM images of the area of abrasive fiber dust accumulation and the brittle-fracture zone shown in Figure 5. We note in Figure 8a that the debris formed at the interface between the two cutting stages consists of twisted fiber material spread over the cutting face. We note from Figure 8b that the brittle-fracture zone presents evenly distributed fiber grains that are severed in the absence of plastic deformation. This verifies the absence of frictional forces, indicating that cracks are extending at a greater speed than that of the blade assembly.

#### 3.3.4. Complete Failure—Tensile-Failure Zone

**Figure 9:**
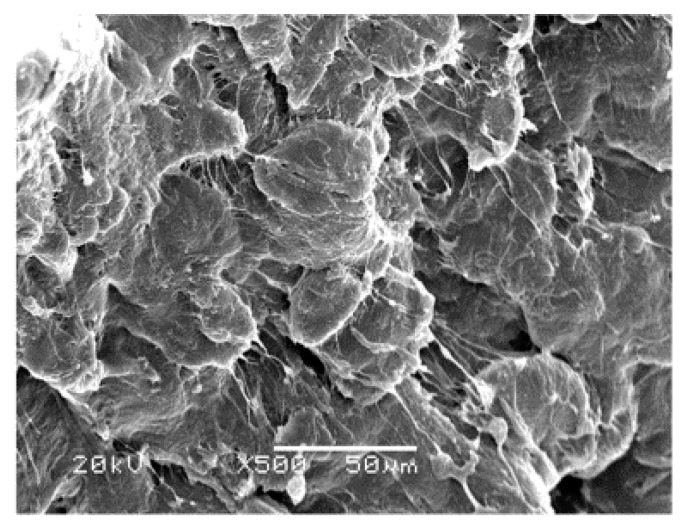
SEM image of the tensile-failure zone shown in Figure 5.

With the occurrence of brittle fracture, the effective cross-sectional area of the fiber rapidly reduces while fiber tension increases. The remaining cross-sectional area is then overwhelmed by the escalating level of fiber tension, and the fiber is completely and suddenly severed in a single catastrophic event. Figure 9 presents a magnified SEM image of the tensile-failure zone shown in Figure 5. We note the formation of strands of fiber material that undergo rapid plastic deformation along the fiber axis prior to failure. The presence of very small tendrils of material indicates that some strands undergo extreme plastic deformation prior to failure. The uneven fracture surface presents no plastic deformation in the cutting direction.

### 3.4. Blade Speed Variation during the Cutting Process

To objectively evaluate variations in the crack extension speed, and to further demonstrate the fiber failure mechanism during fiber cutting, the blade assembly speed was determined from high-speed camera images, and the blade speed was plotted with respect to time over the entire cutting process beginning with the first contact between the cutting blade and the fiber.

**Figure 10:**
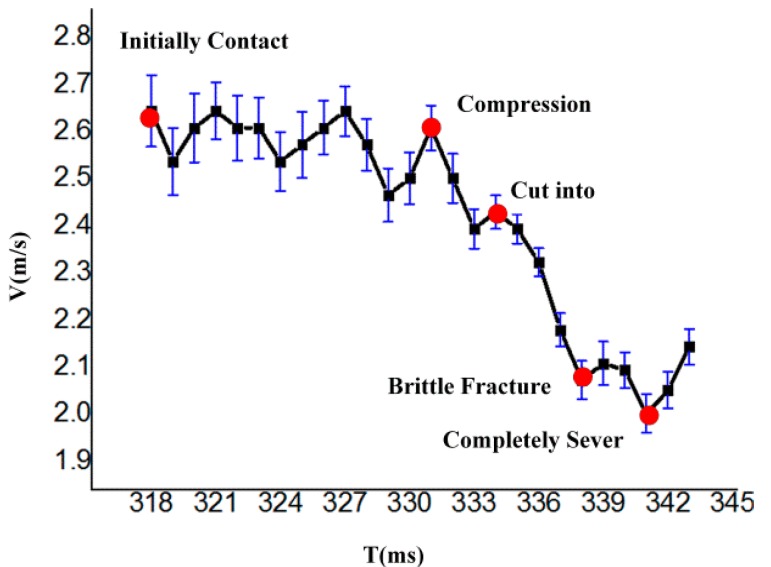
Blade assembly speed curve versus time over the entire cutting process.

As shown in Figure 10, the blade assembly speed fluctuates after first making contact with the fiber, indicating that the fiber initially vibrates prior to the compression stage. PA6 fiber is a type of linearly elastic material, and the fiber undergoes oscillation under impact. In the compression stage, the blade speed continues to fluctuate slightly. With increasing fiber tension, the blade assembly speed begins to decrease, and the fiber undergoes local compression under the normal force applied by the blade tip. The blade speed remains constant for a very short period until the value of σ_B_ exceeds that allowable by the fiber, whereupon the fiber undergoes plastic yielding, initial cracks are created, and the blade begins cutting into the fiber. The blade motion is then subjected to resistance owing to the frictional force between the fiber and the two sides of the blade. Hence, the blade speed decreases rapidly. The cutting resistance continues increasing while the effective cross-sectional area of the fiber rapidly decreases. The failure mechanism enters the brittle-fracture stage when the tensile stress reaches a critical value, and the crack extension speed begins to exceed the blade assembly speed, reducing the frictional force to zero. Hence, the blade assembly speed increases prior to complete fiber failure. The reduced friction and increased blade assembly speed has the effect of sweeping the debris from the blade surface onto the cutting face. After failure, the blade assembly speed naturally increases owing to an absence of resistance.

Analysis shows that the characteristic points on the blade assembly speed curve are fully consistent with the features of fiber fracture morphology obtained from SEM analysis. Future research will focus on the various parameters affecting the fracture failure mechanism, such as the effects of *r*, *m*, and *h*.

## 4. Conclusions

The present study investigated the failure mechanisms of long single PA6 fibers using a custom designed fiber cutting performance test bench. The fracture morphologies were examined by SEM. The analysis revealed that the fiber cutting process can be distinguished according to an initial stage, compression stage, and a fiber failure stage. The fiber failure stage was itself composed of four distinct stages—represented by craze shearing, cutting, brittle fracture, and tensile failure—which are the result of different mechanisms active during the processes of crack initiation, extension, and fracture. The fractographic analysis was further verified by an analysis of the blade assembly speed with respect to time over the entire fracture failure process based on high-speed camera data. The results of fractographic analysis and blade assembly speed were fully consistent. Craze shearing initially occurred owing to the development of a local transverse compressive stress on the fiber at the blade tip, wherein cracks were initiated. The blade assembly speed correspondingly decreased. During the cutting stage, the speed of crack extension was less than the speed of the blade assembly, and a substantial frictional force developed between the two sides of the cutting blade and the two fracture faces, resulting in extensive plastic deformation of the fracture face and a rapidly decreasing blade assembly speed. During the brittle-fracture stage, the speed of crack extension was greater than the speed of the blade assembly, resulting in a rapidly decreasing frictional force and increasing blade assembly speed. As a result, no plastic deformation was observed on the fracture face. Finally, tensile failure occurred when the remaining cross-sectional area was overwhelmed by the escalating level of fiber tension, and the fiber was completely and suddenly severed in a single catastrophic event. This paper provided much needed insight into the cutting mechanisms of widely employed PA6 fibers, and is expected to contribute to an understanding of cutting fracture failure mechanisms useful to the fields of polymer fiber science and engineering.

## Figures and Tables

**Table 1:** Mechanical properties of PA6 fiber.

Chemical Formula	Density (kg/m^3^)	Elastic Modulus (GPa)	Tensile Stress (MPa)	Elongation at Break (%)	Percentage Reduction of Area (%)	Volume Resistivity (Ω·m)
–[NH–(CH_2_)_5_–CO]_n_–	1136	2.4	168	92.3	18.6	1.8 × 10^12^
